# Sinonasal disease among patients with primary ciliary dyskinesia: an international study

**DOI:** 10.1183/23120541.00701-2022

**Published:** 2023-05-22

**Authors:** Yin Ting Lam, Jean-François Papon, Mihaela Alexandru, Andreas Anagiotos, Miguel Armengot, Mieke Boon, Andrea Burgess, Suzanne Crowley, Sinan Ahmed D. Dheyauldeen, Nagehan Emiralioglu, Ela Erdem Eralp, Christine van Gogh, Yasemin Gokdemir, Onder Gunaydın, Eric G. Haarman, Amanda Harris, Isolde Hayn, Hasnaa Ismail-Koch, Bülent Karadag, Céline Kempeneers, Sookyung Kim, Philipp Latzin, Natalie Lorent, Ugur Ozcelik, Charlotte Pioch, Anne-Lise M.L. Poirrier, Ana Reula, Jobst Roehmel, Panayiotis Yiallouros, on behalf of the EPIC-PCD team, Myrofora Goutaki, EPIC-PCD team:, Dilber Ademhan, Mihaela Alexandru, Andreas Anagiotos, Miguel Armengot, Lionel Benchimol, Achim G. Beule, Irma Bon, Mieke Boon, Marina Bullo, Andrea Burgess, Doriane Calmes, Carmen Casaulta, Marco Caversaccio, Nathalie Caversaccio, Bruno Crestani, Suzanne Crowley, Sinan Ahmed D. Dheyauldeen, Sandra Diepenhorst, Nagehan Emiralioglu, Ela Erdem Eralp, Pinar Ergenekon, Nathalie Feyaerts, Gavriel Georgiou, Amy Glen, Christine van Gogh, Yasemin Gokdemir, Myrofora Goutaki, Onder Gunaydın, Eric G. Haarman, Amanda Harris, Isolde Hayn, Simone Helms, Sara-Lynn Hool, Isabelle Honoré, Hasnaa Ismail Koch, Bülent Karadag, Céline Kempeneers, Synne Kennelly, Elisabeth Kieninger, Sookyung Kim, Panayiotis Kouis, Yin Ting Lam, Philipp Latzin, Marie Legendre, Natalie Lorent, Jane S. Lucas, Bernard Maitre, Alison McEvoy, Rana Mitri-Frangieh, David Montani, Loretta Müller, Noelia Muñoz, Heymut Omran, Ugur Ozcelik, Beste Ozsezen, Samantha Packham, Jean-François Papon, Clara Pauly, Charlotte Pioch, Anne-Lise M.L. Poirrier, Johanna Raidt, Ana Reula, Rico Rinkel, Jobst Roehmel, Andre Schramm, Guillaume Thouvenin, Woolf T Walker, Hannah Wilkins, Panayiotis Yiallouros, Ali Cemal Yumusakhuylu, Niklas Ziegahn

**Affiliations:** 1 Institute of Social and Preventive Medicine, University of Bern, Bern, Switzerland; 2 Graduate School for Cellular and Biomedical Sciences, University of Bern, Bern, Switzerland; 3 Assistance Publique-Hôpitaux de Paris, Université Paris-Saclay, Hôpital Bicêtre, Service d'ORL, Le Kremlin-Bicêtre, France; 4 Faculté de Médecine, Université Paris-Saclay, Le Kremlin-Bicêtre, France; 5 Department of Otorhinolaryngology, Nicosia General Hospital, Nicosia, Cyprus; 6 Department of Otorhinolaryngology, and Primary Ciliary Dyskinesia Unit, La Fe University and Polytechnic Hospital, Valencia, Spain; 7 Medical School, Valencia University, Valencia, Spain; 8 Department of Paediatrics, University Hospital, Leuven, Belgium; 9 Primary Ciliary Dyskinesia Centre, Southampton Children's Hospital, Southampton NHS Foundation Trust, Southampton, UK; 10 Paediatric Department of Allergy and Lung Diseases, Oslo University Hospital, Oslo, Norway; 11 Department of Otorhinolaryngology, Head and Neck Surgery, Oslo University Hospital, Oslo, Norway; 12 Faculty of Medicine, University of Oslo, Oslo, Norway; 13 Department of Pediatric Pulmonology, Hacettepe University, School of Medicine, Ankara, Turkey; 14 Department of Pediatric Pulmonology, Marmara University, School of Medicine, Istanbul, Turkey; 15 Department of Otorhinolaryngology – Head and Neck Surgery, Amsterdam UMC, Amsterdam, The Netherlands; 16 Department of Otorhinolaryngology, Hacettepe University, School of Medicine, Ankara, Turkey; 17 Department of Pediatric Pulmonology, Emma Children's Hospital, Amsterdam UMC, Vrije Universiteit, Amsterdam, The Netherlands; 18 Southampton Children's Hospital, University of Southampton, Southampton, UK; 19 Primary Ciliary Dyskinesia Centre, NIHR Respiratory Biomedical Research Centre, University of Southampton, Southampton, UK; 20 Department of Otorhinolaryngology, Head and Neck Surgery, Charité-Universitätsmedizin Berlin, Berlin, Germany; 21 Division of Respirology, Department of Pediatrics, University Hospital Liège, Liège, Belgium; 22 Division of Paediatric Respiratory Medicine and Allergology, Department of Paediatrics, Inselspital, University Hospital, University of Bern, Bern, Switzerland; 23 Department of Respiratory Diseases, University Hospital, Leuven, Belgium; 24 Department of Pediatric Pulmonology, Immunology and Critical Care Medicine, Charité-Universitätsmedizin Berlin, Berlin, Germany; 25 Department of Otorhinolaryngology, University Hospital Liège, Liège, Belgium; 26 Biomedical Sciences Department, CEU-Cardenal Herrera University, Castellón, Spain; 27 Molecular, Cellular and Genomic Biomedicine Group, IIS La Fe, Valencia, Spain; 28 Medical School, University of Cyprus, Nicosia, Cyprus; 29 Pediatric Pulmonology Unit, Hospital “Archbishop Makarios III”, Nicosia, Cyprus; 30 For a list of the EPIC-PCD team members see the Acknowledgements

## Abstract

**Background:**

Sinonasal symptoms are a common feature of primary ciliary dyskinesia (PCD); however, literature about their severity and frequency, particularly during the life course, is scarce. Using baseline data from the Ear, nose and throat (ENT) Prospective International Cohort of PCD patients, we describe sinonasal disease in PCD.

**Methods:**

We included participants who had a routine sinonasal examination during which they completed a symptoms questionnaire. We compared frequency of reported symptoms and examination findings among children and adults, and identified characteristics potentially associated with higher risk of sinonasal disease using ordinal regression.

**Results:**

12 centres contributed 384 participants; median age was 16 years (IQR 9–22), and 54% were male. Chronic nasal problems were the most common feature, reported by 341 (89%). More adults (33; 24%) than children (10; 4%) described hyposmia. Quality of life was moderately affected by rhinosinusitis among 136 participants with completed SNOT-22 questionnaires (median score 31; IQR 23–45). Examinations revealed nasal polyps among 51 of 345 participants (15%) and hypertrophic inferior nasal turbinates among 127 of 341 participants (37%). Facial pain was detected in 50 of 342 participants (15%). Nasal polyps, hypertrophic turbinates, deviated septum and facial pain were found more commonly in adults than children. The only characteristic associated with higher risk of sinonasal disease was age 10 years and older.

**Conclusions:**

Based on our findings, regular sinonasal examinations are relevant for patients with PCD of all ages. There is a need for improved management of sinonasal disease supported by evidence-based guidelines.

## Abstract 2

**This study reinforces the importance of regular sinonasal examinations for patients of all ages with PCD and the need to develop evidence-based sinonasal treatments as part of overall PCD management** https://bit.ly/3lMcgbo

## Introduction

Sinonasal symptoms among patients with primary ciliary dyskinesia (PCD) are as common as lower respiratory symptoms [1, 2]. Often present from birth, rhinitis is one of the first signs of PCD and usually persists throughout life [3–6]. With impaired respiratory ciliary movement and reduced mucociliary clearance, nasal secretions depend only on gravity and airflow transport [1, 7]. Sinonasal problems may manifest with rhinorrhea or blocked nose, facial pain and headaches [8, 9]. With PCD, symptoms are part of daily life, often considered normal, and likely underreported during routine consultations. Sinonasal disease is also characterised by recurrent upper respiratory infections, often leading to chronic rhinosinusitis (CRS). Despite the clinical burden, sinonasal manifestations are frequently neglected, and in many centres, ear-nose-throat (ENT) assessments are not part of routine multidisciplinary PCD care, particularly for adults [10–16]. Since sinuses may function as bacterial reservoirs for pulmonary infections later leading to lung function impairment, sinus infections are often considered only after unsuccessful treatment of pulmonary infections [17–22]. Nasal polyps are common in patients with PCD and are found in 15–30% of cases compared to a prevalence of 3–4% in the general population [6, 23]. Other sinonasal manifestations among patients with PCD include hypoplasia or agenesis of paranasal sinuses [8, 24].

The few published studies on sinonasal manifestations in PCD are mostly retrospective, include small numbers (20–60) of participants who are primarily children, and obtain data from chart reviews where symptoms were collected in a nonstandard way [2, 25]. Little is known about progression of sinonasal disease with age or with increased frequency of sinonasal symptoms. We aimed to describe the prevalence of patient-reported sinonasal symptoms and sinonasal examination findings among children and adults with PCD and identify possible risk factors associated with sinonasal disease.

## Methods

### Study design and population

Our study analyses cross-sectional baseline data from the ENT Prospective International Cohort of Patients with PCD (EPIC-PCD), the first PCD cohort focused on upper airway disease manifestations [26]. We set up EPIC-PCD in February 2020 to follow PCD patients at their routine ENT consultations. Participants did not undergo additional testing for our study purposes. EPIC-PCD is hosted at the University of Bern (clinicaltrials.gov identifier: NCT04611516). For our collaborative study, 12 participating centres (Amsterdam, Ankara, Berlin, Bern, Cyprus, Istanbul, Leuven, Liège, Oslo, Paris, Southampton and Valencia) in 10 countries contributed data. For our analysis, we included data entered in the database by 31 July 2022 for participants with PCD of all ages who underwent ENT examinations and completed symptoms questionnaires at the same visit or within 2 weeks.

We received ethical approval from all participating centres and human research ethics committees in accordance with local legislation. We obtained informed consent or assent from either participants or parents or caregivers of participants 14 years or younger. Our report conforms with the Strengthening the Reporting of Observational studies in Epidemiology (STROBE) statement [27].

### Patient-reported symptoms and quality of life

For collecting patient-reported symptoms, we used the disease-specific FOLLOW-PCD questionnaire (version 1.0), which is part of the FOLLOW-PCD form developed to collect clinical information for research and clinical follow-up in a standardised way [28]. There are age-specific versions of the FOLLOW-PCD questionnaire for adults, adolescents 14–17 years, and parents or caregivers of children with PCD 14 years and younger. The FOLLOW-PCD questionnaire is available in languages of participating centres. Sinonasal symptoms questions ask about frequency and characteristics of symptoms during the past 3 months, specifically focusing on chronic nasal symptoms, snoring and headaches, as well as more frequent ENT symptoms during the past 12 months. Symptom frequency options included daily, often, sometimes, rarely and never (five-point Likert scale). Lifestyle questions asked about smoking exposure and living conditions during the past 12 months. Depending on available response categories, we recoded missing answers as “unknown”, “no” or “never”.

Based on local protocols, if distributed during the clinic visit we also collected information about quality of life (QoL) using the Sino-Nasal Outcome Test (SNOT-22) [29]. SNOT-22 is a validated CRS health-related QoL outcome measure. Participants give CRS-related items scores of 0–5 each, ranging from “no problem” to “problem as bad as it can be”. In total, SNOT-22 scores range between 0 and 110, corresponding to a mild (0–20), moderate (21–50) or severe (≥51) effect of CRS on QoL.

### Sinonasal examinations

The EPIC-PCD is nested in routine care and follows participants at their usual ENT consultations. Performed by an ENT specialist according to local protocols, routine ENT consultations included clinical sinonasal examinations by nasal endoscopy or anterior rhinoscopy if tolerated by the participant. Examination findings were recorded in a standardised way using the ENT examination module of the FOLLOW-PCD form [28]. We recorded the proportion of the total nasal cavity volume occupied by nasal polyps using a semi-quantitative measure – the Lildholdt score – described as “partially blocking” (Lildholdt scores 1–2) and “fully blocking” (Lildholdt score 3) [30]. We recorded, reported and present missing information from sinonasal examinations as missing.

### Diagnosis and other clinical information from charts

Participants were diagnosed according to European Respiratory Society (ERS) guidelines [31]. Positive PCD diagnosis was confirmed by presence of hallmark ultrastructural defects seen in transmission electron microscopy (TEM) or by identification of bi-allelic pathogenic mutations in PCD genes [32]. Participants with low nasal nitric oxide and high-speed video microscopy analysis findings indicative of PCD, possibly in combination with other diagnostic tests supporting diagnosis, were considered to have a highly likely PCD diagnosis. Remaining participants were categorised as probable PCD and had at least one diagnostic test result supporting diagnosis in addition to symptoms consistent for PCD. These patients were treated as PCD patients at respective PCD centres and usually did not have all diagnostic tests performed (supplementary table S1). We collected data on laterality defects from medical records and, when it was available, past medical history information, particularly about neonatal rhinitis. Lastly, in addition to the basic dataset, some participating centres contributed information on prescribed sinonasal management. We entered all collected data in the study database, which uses the Research Electronic Data Capture (REDCap) software, based on the FOLLOW-PCD form [28].

### Statistical analysis

We described characteristics of the population, patient/parent-reported sinonasal symptoms and sinonasal examination findings for the total population and separately for age groups 0–6, 7–14, 15–30, 31–50 and 50 years and older. For continuous variables, we used median and interquartile range (IQR); for categorical variables, we used numbers and proportions, calculating Wilson 95% confidence intervals (CI) for proportions. We compared differences between age groups using Pearson's Chi-square, Wilcoxon rank-sum and Kruskal–Wallis rank tests. As sensitivity analysis and to test the robustness of our findings, we described separately patient/parent-reported sinonasal symptoms and sinonasal examination findings in the subgroup of patients with positive PCD diagnosis according to the ERS guidelines. We created a composite outcome variable for sinonasal disease consisting of three variables: patient-reported headache while bending down as a proxy for sinusitis, ENT examination findings of nasal polyps and facial pain. Each of them scored either 0 (absence) or 1 (presence). Total scores ranged from 0 to 3. We assessed factors possibly associated with sinonasal disease such as age, age of diagnosis, sex, study centre, smoking status of either active or passive smoke exposure, and season when ENT consultations occurred in a multivariable ordinal logistic regression model. We chose factors based on clinical importance and data availability. There was collinearity of age and age of diagnosis so it was not possible to include both in our main model; separate models showed similar results so we included age. After exploring linear and nonlinear effects of age as continuous variable, we chose to include age by decades in the final model. We excluded study centre from the full model due to restricted sample size; however, we conducted sensitivity analyses with study centre alone and with age. Lastly, among a subgroup of participants with available TEM results, we repeated the model including age and category of ciliary ultrastructural defect to study if ciliary ultrastructural defect was associated with risk for sinonasal disease. We performed all analyses with Stata version 15 (StataCorp LLC, College Station, TX, USA).

## Results

### Study population

**FIGURE 1:**
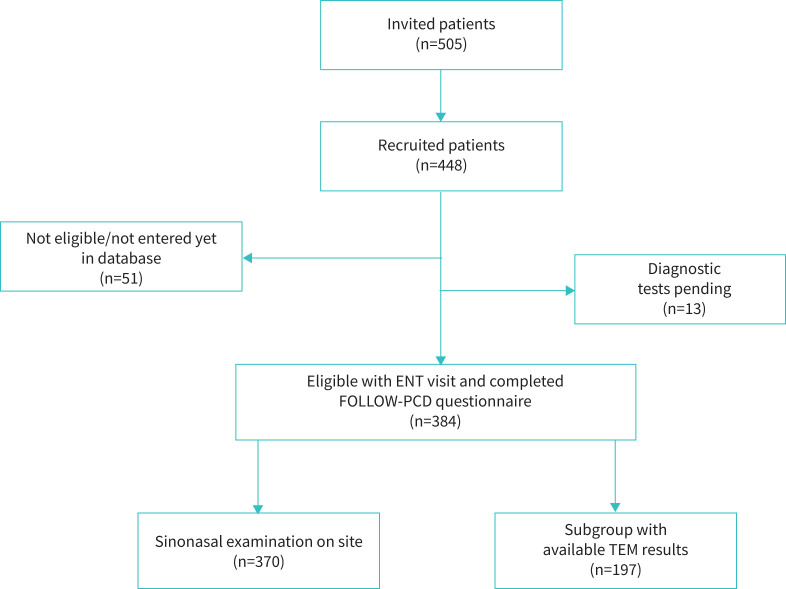
Flowchart of EPIC-PCD (ENT Prospective International Cohort of Patients with Primary Ciliary Dyskinesia) study population. ENT: ear-nose-throat; PCD: primary ciliary dyskinesia; TEM: transmission electron microscopy.

By the end of July 2022, 448 (89%) of 505 invited patients with PCD enrolled in the EPIC-PCD cohort (figure 1). Of these participants, 384 (54% male) with median age 16 years (IQR 9–22) entered in the database fulfilled eligibility criteria for ENT consultation and completed a FOLLOW-PCD questionnaire at the same visit or within 2 weeks (table 1). 247 (64%) participants were children, 137 (36%) adults and 134 (35%) had situs inversus totalis. With regard to participant diagnostic status, 257 (67%) had a PCD positive diagnosis based on ERS guidelines [31] with a bi-allelic PCD-causing mutation or a hallmark defect identified by TEM (supplementary tables S1–2); 59 (15%) had highly likely PCD. The remaining 68 (18%) participants had probable PCD diagnosed with typical symptoms and with at least one pathological test indicating PCD.

**TABLE 1:** Characteristics of EPIC-PCD participants, overall and by age group (n=384)

	**Total**	**Age 0–6 years**	**Age 7–14 years**	**Age 15–30 years**	**Age 31–50 years**	**Age >50 years**	**p-value^#^**
**Participants**	384 (100)	44 (100)	122 (100)	153 (100)	42 (100)	23 (100)	
**Age years**	16 (9–22)	4 (2–5)	10 (8–12)	18 (16–21)	38 (34–43)	57 (56–62)	
**Male sex**	206 (54)	23 (52)	69 (57)	79 (52)	24 (57)	11 (48)	0.875
**Age at PCD diagnosis**	9 (4–17)	1 (1–2)	6 (2–8)	13 (9–17)	34 (29–36)	51 (43–55)	
**Consanguinity**							0.001
** **Yes	115 (30)	7 (15)	44 (36)	49 (32)	11 (26)	4 (17)	
** **No	130 (34)	13 (30)	35 (29)	60 (39)	19 (45)	3 (13)	
** **Not reported	139 (36)	24 (55)	43 (35)	44 (29)	12 (29)	16 (70)	
**Situs**							<0.001
** **Situs inversus totalis	134 (35)	25 (57)	42 (34)	56 (37)	7 (17)	4 (17)	
** **Situs ambiguous	4 (1)	0 (0)	1 (1)	3 (2)	0 (0)	0 (0)	
** **Situs solitus	242 (63)	18 (41)	79 (65)	94 (61)	32 (76)	19 (83)	
** **Not reported	4 (1)	1 (2)	0 (0)	0 (0)	3 (7)	0 (0)	
**Cardiovascular malformation present**							0.093
** **Yes	33 (9)	7 (16)	10 (8)	14 (9)	2 (5)	0 (0)	
** **No	285 (74)	30 (68)	98 (80)	110 (72)	32 (76)	15 (65)	
** **Not reported	66 (17)	7 (16)	14 (12)	29 (19)	8 (19)	8 (35)	
**Neonatal rhinitis**							0.145
** **Yes	123 (32)	15 (34)	46 (38)	48 (31)	12 (29)	2 (9)	
** **No	83 (22)	7 (16)	23 (19)	38 (25)	11 (26)	4 (17)	
** **Not reported	178 (46)	22 (50)	53 (43)	67 (44)	19 (45)	17 (74)	
**Neonatal cough**							0.001
** **Yes	125 (33)	15 (34)	42 (34)	57 (37)	10 (24)	1 (4)	
** **No	86 (22)	3 (7)	28 (23)	38 (25)	14 (33)	3 (13)	
** **Not reported	173 (45)	26 (59)	52 (43)	58 (38)	18 (43)	19 (83)	
**Neonatal respiratory distress**							0.489
** **Yes	175 (46)	26 (59)	57 (46)	20 (48)	20 (47)	8 (35)	
** **No	139 (36)	11 (25)	46 (38)	15 (36)	15 (36)	8 (35)	
** **Not reported	70 (18)	7 (16)	19 (16)	7 (17)	7 (17)	7 (30)	
**Active smoking**							<0.001
** **Yes, daily	4 (1)	NA	NA	3 (2)	0 (0)	1 (4)	
** **Yes, rarely	5 (1)	NA	NA	3 (2)	1 (2)	1 (4)	
** **Ex-smoker	15 (4)	NA	NA	2 (1)	8 (19)	5 (22)	
** **Never-smoker	183 (48)	NA	NA	137 (90)	31 (74)	15 (66)	
** **Not reported	177 (46)	NA	NA	8 (5)	2 (5)	1 (4)	
**Smoking in household**							0.001
** **Yes	66 (17)	6 (14)	22 (18)	33 (22)	3 (7)	2 (9)	
** **No	254 (66)	34 (77)	92 (75)	86 (56)	27 (64)	15 (65)	
** **Not reported	64 (17)	4 (9)	8 (7)	34 (22)	12 (29)	6 (26)	

Data are presented as n (%) or median (IQR). EPIC-PCD: Ear-nose throat Prospective International Cohort of patients with Primary Ciliary Dyskinesia; NA: not applicable (age-specific questionnaire version does not include question on active smoking in this age category). ^#^: Chi-square test of independence.

### Patient-reported symptoms and QoL

Chronic nasal symptoms were very common, with most (341; 89%) participants reporting nasal symptoms during the past 3 months (table 2). Over half of participants (198; 52%) reported chronic nasal symptoms daily or often, which “persisted all the time” for 140 participants (41%). Rhinorrhoea was the most commonly (306; 90%) reported nasal symptom, although nasal discharge colour varied. Some participants reported anosmia or hyposmia (43; 13%) and nearly half (185; 48%) reported snoring. Of those participants reporting snoring, 42 snored almost every night (23%), and 82 even during periods when they did not have colds (44%). Most participants reported headaches (238; 62%), which for some occurred mainly while bending down (42; 11%). Far fewer participants (26; 7%) suffered from migraines. More ENT symptoms were reported during December when compared with other months. In comparison with children, more adults reported anosmia or hyposmia (24% *versus* 4%, p<0.001), headaches (73% *versus* 56%, p<0.114) and migraines (15% *versus* 2%, p<0.001). We did not find other differences with patient-reported sinonasal symptoms by age or sex. Only 24 (6%) participants reported no sinonasal symptoms (supplementary figure S1). Results of reported symptoms were similar in the subgroup of patients with positive diagnosis (supplementary table S3).

**TABLE 2:** Upper respiratory symptoms of past 3 months reported by EPIC-PCD participants, overall and by age group (n=384)

	**Total**	**Age 0–6 years**	**Age 7–14 years**	**Age 15–30 years**	**Age 31–50 years**	**Age >50 years**	**p-value^#^**
**Participants**	384 (100)	44 (100)	122 (100)	153 (100)	42 (100)	23 (100)	
**Nasal symptoms**							0.199
** **Daily/often	198 (52)	19 (43)	65 (53)	70 (46)	28 (67)	16 (70)	
** **Sometimes/rarely	143 (37)	18 (41)	45 (37)	63 (41)	11 (26)	6 (26)	
** **Never	43 (11)	7 (16)	12 (10)	20 (13)	3 (7)	1 (4)	
**Nasal symptoms persisting all the time** ^¶^	140 (41)	17 (50)	45 (41)	49 (37)	21 (54)	8 (25)	0.535
**Type of nasal symptoms^+^**							
** **Rhinorrhoea	306 (90)	34 (92)	96 (87)	120 (90)	35 (90)	21 (91)	0.661
** **Blocked nose	232 (68)	17 (46)	78 (71)	94 (71)	31 (79)	12 (38)	0.883
** **Sneezing	74 (22)	5 (14)	18 (15)	31 (19)	9 (10)	11 (18)	0.380
** **Anosmia/hyposmia	43 (13)	0 (0)	7 (6)	13 (10)	13 (33)	10 (45)	<0.001
**Colour of nasal discharge in case of rhinorrhoea** ^§^							
** **Clear	61 (20)	9 (27)	16 (17)	26 (22)	6 (17)	4 (19)	0.729
** **White	57 (19)	6 (18)	22 (23)	19 (17)	6 (17)	4 (19)	0.856
** **Yellow	103 (34)	10 (29)	32 (33)	44 (37)	12 (34)	5 (24)	0.676
** **Green	74 (24)	9 (26)	25 (26)	26 (22)	8 (23)	6 (28)	0.977
** **Mixed with blood	11 (4)	0 (0)	1 (1)	5 (4)	3 (9)	2 (10)	0.173
**Snoring**							0.003
** **Daily/often	45 (12)	5 (11)	14 (11)	10 (6)	11 (26)	5 (22)	
** **Sometimes/rarely	140 (36)	20 (45)	49 (40)	47 (31)	14 (33)	10 (43)	
** **Never/not reported	199 (52)	19 (43)	59 (48)	96 (63)	17 (40)	8 (35)	
**Periods of snoring** ^ƒ^							0.101
** **Almost every night	42 (23)	5 (20)	13 (21)	8 (14)	10 (40)	6 (40)	
** **Only during colds	45 (24)	9 (36)	17 (27)	11 (19)	6 (24)	2 (13)	
** **Sometimes also without colds	82 (44)	8 (32)	29 (46)	34 (60)	6 (24)	5 (34)	
** **Not reported	16 (9)	3 (12)	4 (6)	4 (7)	3 (12)	2 (13)	
**Headache**							<0.001
** **Daily/often	48 (12)	0 (0)	14 (11)	20 (13)	8 (19)	6 (26)	
** **Sometimes/rarely	190 (50)	9 (20)	53 (43)	90 (59)	28 (67)	10 (43)	
** **Never/not reported	146 (38)	35 (80)	55 (45)	43 (28)	6 (14)	7 (30)	
**Headache when bending down**	42 (11)	1 (2)	6 (5)	28 (18)	5 (12)	2 (9)	0.002
**Migraines**							<0.001
** **Yes	26 (7)	0 (0)	3 (3)	10 (7)	6 (14)	7 (30)	
** **No	358 (93)	44 (100)	119 (97)	143 (93)	36 (86)	16 (70)	
**SNOT–22 completed**	136 (35)	14 (32)	27 (22)	54 (35)	27 (64)	14 (61)	<0.001
**SNOT–22 score**	31 (23–45)	25 (15–36)	28 (20–45)	29 (17–38)	36 (26–51)	63 (35–79)	<0.001^##^

Data are presented as n (%) or median (IQR). Sino-Nasal Outcome Test-22 (SNOT–22) questionnaire on chronic rhinosinusitis related items scored 0–5 (“No problem” to “Problem as bad as it can be”), total score range 0–110, mild 0–20, moderate 21–50, severe ≥51. EPIC-PCD: Ear-nose throat Prospective International Cohort of Patients with Primary Ciliary Dyskinesia. ^#^: Chi-square test of independence; ^¶^: among 341 people with chronic nasal symptoms; ^+^: among 341 people with chronic nasal symptoms, categories are not exclusive; ^§^: among 306 people with rhinorrhoea, categories are not exclusive; ^ƒ^: among 185 people with snoring; ^##^: Kruskal–Wallis test.

In total, 136 (35%) participants completed SNOT-22 questionnaires, who were most commonly adults. The median score was 31 (IQR 23–45), reflecting a moderate effect of CRS symptoms on QoL (table 2). Median SNOT-22 scores were higher with age; we observed the most severe effect on QoL (63; IQR 35–79) among participants aged 50 years and older (p<0.001), and among participants with daily nasal symptoms (supplementary figure S1).

### Sinonasal clinical examinations

We excluded 14 of 384 study participants from our analysis of sinonasal examination findings for remote sinonasal consultations without sinonasal examinations. Among the remaining 370 participants, recording of sinonasal findings from sinonasal examinations was incomplete for some (table 3). For 159 (43%) participants, the nose appeared blocked, while nasal discharge was mainly serous (85; 31%) or sero-mucous (121; 44%). Abnormal nasal mucosa findings were recorded for 165 (45%), specifically mucosal oedema for 104 (28%) participants. Nasal polyps were assessed in 345 participants, and identified in 51 (14%; median age 20 years; IQR 14–36) participants with 24 (47%) located bilaterally. Of the 51 participants, 38 (74%) had nasal polyps either partially (38; 74%) or fully (9; 18%) blocking nasal passages. Nasal turbinates were hypertrophic in 127 participants (34%), and 117 participants had deviated septum (31%). 50 participants had facial pain (13%) at examination. When compared with children, more adults had nasal polyps, hypertrophic turbinates, deviated septum and facial pain (all p<0.003). We did not find differences according to sex. Clinical findings were similar in the subgroup of patients with positive PCD diagnosis (supplementary table S4).

**TABLE 3:** Sinonasal examination results of EPIC-PCD participants, overall and by age group (n=370)

	**Total**	**Age 0–6 years**	**Age 7–14 years**	**Age 15–30 years**	**Age 31–50 years**	**Age >50 years**	**p-value^#^**
**ENT consultations on site**	370 (100)	38 (100)	116 (100)	151 (100)	42 (100)	23 (100)	
**Nose appearance**							0.044
** **Normal	203 (55)	23 (60)	72 (62)	84 (56)	15 (36)	9 (39)	
** **Blocked	159 (43)	14 (37)	42 (36)	65 (43)	26 (62)	12 (52)	
** **Not recorded	8 (2)	1 (3)	2 (2)	2 (1)	1 (2)	2 (9)	
**Nasal discharge present**							0.746
** **Yes	276 (75)	28 (74)	89 (77)	108 (72)	33 (79)	18 (78)	
** **No	86 (23)	9 (24)	25 (21)	41 (27)	7 (17)	4 (17)	
** **Not recorded	8 (2)	1 (3)	2 (2)	2 (1)	2 (5)	1 (4)	
**Type of nasal discharge** ^¶^							0.833
** **Serous	85 (31)	10 (36)	29 (33)	32 (29)	8 (24)	6 (33)	
** **Sero-mucous	121 (44)	11 (39)	42 (47)	45 (42)	16 (49)	7 (39)	
** **Muco-purulent	60 (22)	6 (21)	16 (18)	26 (24)	8 (24)	4 (22)	
** **Mixed with blood	3 (1)	1 (4)	0 (0)	1 (1)	0 (0)	1 (6)	
** **Not recorded	7 (3)	0 (0)	2 (2)	4 (4)	1 (3)	0 (0)	
**Nasal mucosa**							0.021
** **Abnormal	165 (45)	13 (34)	52 (45)	65 (43)	21 (50)	14 (61)	
** **Normal	194 (52)	21 (55)	61 (52)	85 (56)	20 (48)	7 (30)	
** **Not recorded	11 (3)	4 (11)	3 (3)	1 (1)	1 (2)	2 (9)	
**Nasal polyps**							0.001
** **Yes	51 (14)	2 (5)	11 (10)	21 (14)	12 (28)	5 (22)	
** **No	294 (79)	28 (74)	96 (83)	125 (83)	28 (67)	17 (74)	
** **Not assessed	25 (7)	8 (21)	9 (8)	5 (3)	2 (5)	1 (4)	
**Nasal polyps size^+,^** ^§^							0.837
** **Fully blocking	9 (18)	1 (50)	3 (27)	3 (14)	2 (17)	0 (0)	
** **Partially blocking	38 (74)	1 (50)	7 (64)	16 (76)	9 (75)	5 (100)	
** **Not assessed	4 (8)	0 (0)	1 (9)	2 (9)	1 (8)	0 (0)	
**Bilaterally^+,^** ^§^							0.436
** **Fully blocking	4 (8)	0 (0)	2 (18)	0 (0)	2 (17)	0 (0)	
** **Partially blocking	16 (31)	1 (50)	3 (27)	7 (33)	2 (17)	3 (60)	
** **Not recorded	31 (61)	1 (50)	6 (55)	14 (67)	8 (67)	2 (40)	
**Unilaterally^+,^** ^§^							0.448
** **Fully blocking	6 (12)	1 (50)	1 (9)	2 (9)	2 (17)	0 (0)	
** **Partially blocking	27 (53)	0 (0)	7 (64)	9 (43)	7 (58)	4 (80)	
** **Not recorded	18 (35)	1 (50)	3 (27)	10 (48)	3 (25)	1 (20)	
**ENT** **consultations on site**	370 (100)	38 (100)	116 (100)	151 (100)	42 (100)	23 (100)	
**Inferior nasal turbinates**							0.003
** **Normal	211 (57)	21 (55)	61 (53)	97 (64)	20 (48)	12 (52)	
** **Hypertrophy	127 (34)	13 (34)	46 (40)	45 (30)	15 (36)	8 (35)	
** **Atrophy	3 (1)	0 (0)	0 (0)	0 (0)	3 (7)	0 (0)	
** **Not recorded	29 (8)	4 (11)	9 (7)	9 (6)	4 (9)	3 (13)	
**Deviated nasal septum**							<0.001
** **Yes	112 (30)	2 (5)	26 (22)	62 (41)	12 (29)	10 (43)	
** **Bulging forward	5 (1)	1 (3)	0 (0)	1 (1)	2 (5)	1 (4)	
** **No	230 (60)	28 (74)	84 (72)	81 (54)	27 (64)	10 (43)	
** **Not recorded	23 (6)	7 (18)	6 (5)	7 (5)	1 (2)	2 (9)	
**Facial pain or sensitivity**							<0.001
** **Yes	50 (13)	0 (0)	8 (7)	21 (14)	12 (29)	9 (39)	
** **No	292 (79)	27 (71)	102 (88)	123 (81)	27 (64)	13 (56)	
** **Not recorded	28 (8)	11 (29)	6 (5)	7 (5)	3 (7)	1 (4)	

Data are presented as n (%). EPIC-PCD: Ear-nose-throat Prospective International Cohort of patients with Primary Ciliary Dyskinesia; ENT: ear, nose and throat; ^#^: Chi-square test of independence; ^¶^: among 276 people with nasal discharge; ^+^: among 51 participants with nasal polyps; ^§^: nasal polyps described as partially blocking or with Lildholdt score 1 or 2, fully blocking or with Lildholdt score 3.

### Information on management of upper airways

At baseline, 76 (19%) participants had hospitalisations since previous consultation, yet it was unattributed to upper respiratory infections (supplementary table S5). A small proportion of participants (20; 4%) underwent elective operations, nine of them for sinonasal complications (53%), during this period. Nearly one-quarter (82; 21%) of 331 participants were prescribed nasal corticosteroids, most commonly for year-round use (69; 84%). Out of 282 participants, the most common relevant nasal corticosteroid instruction involved regular nose blowing (164; 58%); out of 297 participants, instructions commonly involved nasal rinsing (187; 63%); both instructions recommended mostly year-round use. Lastly, 46 of 258 participants (18%) were prescribed upper airway nebulisation prescriptions with isotonic saline (17; 37%), hypertonic saline (22; 48%) or other medication (6; 13%). Most commonly (43; 93%) these were prescribed for year-round use.

### Factors associated with sinonasal disease

**FIGURE 2:**
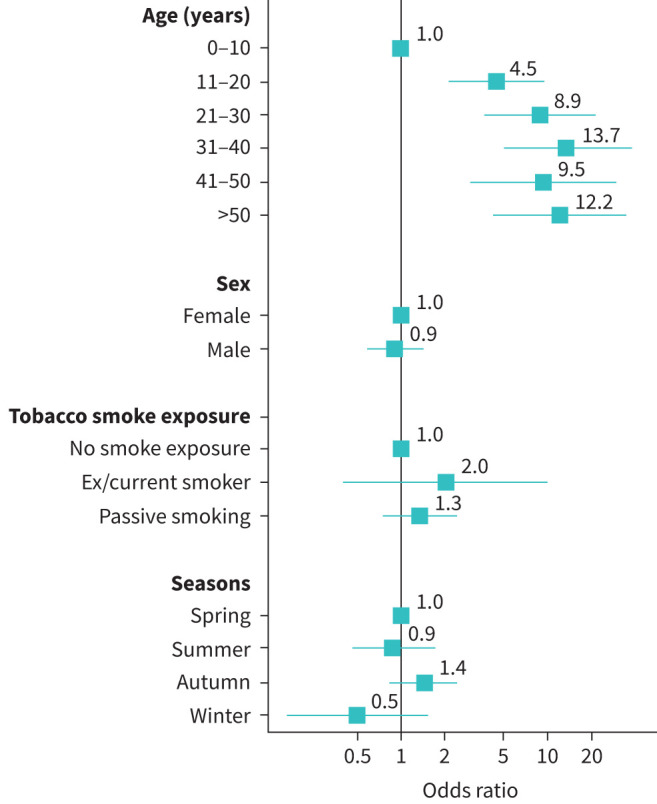
Factors associated with sinonasal disease in EPIC-PCD participants (n=384). Sinonasal disease defined by composite outcome score consisting of three variables: patient-reported headache while bending down as a proxy for sinusitis, and ear, nose and throat examination findings of nasal polyps and facial pain. Odds ratio indicated by squares and 95% confidence intervals by horizontal lines. EPIC-PCD: Ear-nose-throat Prospective International Cohort of Patients with Primary Ciliary Dyskinesia.

We found age 10 years and older associated with higher risk of sinonasal disease; this association was greater when comparing participants aged 31–40 years with those aged 0–10 years (odds ratio (OR): 13.73, 95% CI: 4.96–37.95). Even after accounting for age, risk also differed based on study centre (supplementary table S6). We did not find associations with sex, tobacco smoke exposure or season when consultations took place (figure 2). In the subgroup analyses of 197 participants with available TEM results (supplementary table S2), we found no association between ciliary ultrastructural defect class and risk of sinonasal disease (supplementary figure S3), except of an increasing trend for higher risk of sinonasal disease in participants with central complex defects (OR: 2.1, 95% CI: 0.61–7.04) and other non-hallmark defects (OR: 1.9, 95% CI: 0.68–5.38).

## Discussion

Our study benefitted from data from the first prospective, multicentre, international ENT cohort of patients with PCD. Even though we performed our study during the COVID-19 pandemic, with much lower prevalence of viral infections, most participants reported chronic nasal symptoms, most commonly rhinorrhea. Our results showed sinonasal symptoms, and clinical examination findings indicated chronic inflammation that was also more common with increasing age. Overall, QoL, as measured by SNOT-22, was moderately affected by CRS (median score 31; IQR 23–45). Anterior rhinoscopic or endoscopic findings, such as nasal polyps, hypertrophic turbinates and deviated septum, as well as facial pain at examination, were more commonly found among adults than children. We found the risk of sinonasal disease increased with age and was associated with study centre.

### Strengths and limitations

Our study's main strength includes our use of data from a large, prospective, international cohort with an overall recruitment rate of 89%. We are the first to describe patient-reported sinonasal symptoms and sinonasal examination findings obtained during the same consultation for PCD. Another strength is our use of FOLLOW-PCD, which allowed standardised records of disease-specific information and comparisons between participating centres. We excluded participants if their data were not yet entered in the study database or they did not meet the eligibility criteria. We have no reason to think exclusions were not random or affected the representation of our study population, but participants with more sinonasal symptoms might be more willing to join EPIC-PCD when invited. We expect small risks of recall bias for patient-reported symptoms since questionnaires ask about the last 3 months. However, these symptoms are unspecific and part of participants’ daily life, so they might be underreported. Particularly, assessing anosmia or hyposmia among young children is difficult and possibly underreported by parents. Mostly, adult participants completed the SNOT-22 questionnaires, which is expected since it is only validated for adults and not used at all participating centres. We still do not know if the score sufficiently captures or underestimates effects from CRS on QoL among children. Another reason might be that the prevalence of CRS increases with age among adults in the general population; in particular CRS without nasal polyps is more prevalent among adults 40 years or younger and CRS with nasal polyps is more evident among adults 40 years or older [23]. Although our cohort was set up at the beginning of the COVID-19 pandemic, based on data from the COVID-PCD study SARS-CoV-2 infections were infrequent and caused generally mild to moderate symptoms among people with PCD [33], probably due to participants' careful shielding behaviours [34]. It is likely that the shielding behaviours of people with PCD led to fewer infections, resulting in lower prevalence and underestimation of sinonasal problems, yet almost all participants reported nasal symptoms.

### Comparison with other studies

Previous studies of upper respiratory symptoms also showed that ENT symptoms are common among people with PCD; however, ENT symptom definitions varied, making comparisons difficult. For instance, in a prospective study using a nationwide survey based on the FOLLOW-PCD questionnaire in Switzerland, 70 (95%) of 74 participants reported chronic nasal symptoms with rhinorrhoea (65%), blocked nose (55%) or anosmia (38%) [9]. In comparison with this study, the older population of the Swiss study or differences in upper airway management could explain the higher prevalence of anosmia. Differences in upper airway management among participating study centres might explain the differences in the risk of sinonasal disease we found, but in-depth comparisons require more detailed data. Similar to our findings, a prospective study in North America described CRS among 47 children with nasal polyps (3; 6%) and snoring (23; 49%), and a mean SNOT-22 score of 36.4 [35]. In a retrospective study in France, 63 of 64 adults reported sinonasal problems along with pathological nasal endoscopic findings [36], which is similar to our adult population's chronic nasal problems. In the same study, there was no correlation of ENT disease severity with ciliary ultrastructural defects. In another study assessing 39 adults with PCD and CRS in Italy, 59% had nasal polyps and more severely affected QoL, as measured by SNOT-22 score, than those without nasal polyps [37]. Their findings were more severe than among our population, probably because we included children and more young adults. A study including 67 adults with PCD in Japan supports our finding that nasal polyps were observed more frequently with increasing age [38]. Although we did not observe this, higher odds of having CRS have been described for tobacco smoke exposure in the general population. However, our population reported a small number of participants exposed to tobacco, particularly active smoking [39]. Our results showed an increasing trend for higher risk of sinonasal disease in participants with central complex defects and other non-hallmark defects. According to the literature, these defects are usually not associated with more severe disease; however a large international study also reported that children and young adults with central complex defects had the worst baseline lung function compared to all other participants apart from those with microtubular disorganisation [22].

### Conclusion

We found that sinonasal problems persist throughout life among people with PCD. In particular, more adults had nasal polyps and reported anosmia or hyposmia, showing that complications of CRS increase with age, possibly as a result of ongoing chronic inflammation. Overall, patients reported a moderate effect of their sinonasal problems on QoL. A possible explanation is that they likely grew accustomed to the symptoms and their effects, therefore underreporting limitations in QoL. Although most participants frequently reported sinonasal symptoms, not all were prescribed sinonasal treatment or management, which could be due to patient underreporting or lack of standardised care and evidence-based PCD management guidelines for upper airways. Our study reinforces the importance of regular sinonasal examinations for PCD patients of all ages and the need to develop evidence-based sinonasal treatments as part of the overall PCD management.

## Supplementary material

10.1183/23120541.00701-2022.Supp1 **Please note:** supplementary material is not edited by the Editorial Office, and is uploaded as it has been supplied by the author.
Supplementary material 00701-2022.SUPPLEMENT
