# Supplementary material for: Sinonasal disease among patients with primary ciliary dyskinesia: an international study
Source: ERJ Open Res. 2023 May 22;9(3):00701-2022. doi: 10.1183/23120541.00701-2022 (PMC10204851; doi:10.1183/23120541.00701-2022)
Supplement: Supplementary file 1 [file 00701-2022.SUPPLEMENT.pdf]

Supplementary data "Sinonasal disease among patients with primary ciliary dyskinesia – an international study"

Table S1: Test results supporting PCD diagnosis among EPIC-PCD participants (N=384)

Diagnostic tests indicative of PCD	N (%)
**PCD positive**	
Bi-allelic pathogenic mutation or hallmark TEM defect⁺	257 (67)
**PCD highly likely**	
Low nNO* and HSVA findings consistent with PCD	46 (12)
Low nNO*, HSVA findings consistent with PCD and non-hallmark TEM defect#	6 (1.6)
Low nNO*, HSVA findings consistent with PCD and single allele pathogenic mutation(s)	3 (0.8)
Low nNO*, HSVA and IF findings consistent with PCD, and single allele pathogenic mutation(s)	2 (0.5)
Low nNO*, HSVA and IF findings consistent with PCD	2 (0.5)
**PCD probable**	
HSVA findings consistent with PCD and single allele pathogenic mutation(s)	5 (1.3)
HSVA and IF findings consistent with PCD, and single allele pathogenic mutation(s)	1 (0.3)
HSVA findings consistent with PCD and non-hallmark TEM defect#	2 (0.5)
HSVA and IF findings consistent with PCD	1 (0.3)
Low nNO* and single allele pathogenic mutation(s)	5 (1.3)
Low nNO* and non-hallmark TEM defect#	4 (1)
Low nNO* and IF findings consistent with PCD	3 (0.8)
Non-hallmark TEM defect# and single allele pathogenic mutation(s)	2 (0.5)
HSVA findings consistent with PCD	23 (6)
Low nNO*	7 (1.8)
IF findings consistent with PCD	2 (0.5)
Non-hallmark TEM defect#	1 (0.3)
Single allele pathogenic mutation(s)	12 (3)

EPIC-PCD: Ear-nose throat prospective international cohort of patients with primary ciliary dyskinesia, TEM: transmission electron microscopy. ODA: outer dynein arm. IDA: inner dynein arm. nNO: nasal nitric oxide. HSVA: high-speed video microscopy analysis. IF: immunofluorescence

Results presented as N and column %. Diagnostic categories "PCD positive" and "PCD highly likely" according to ERS guidelines for diagnosis of PCD (Lucas et al)

⁺Hallmark TEM defects: ODA, ODA and IDA, microtubular disorganisation and IDA defects (based on BEAT-PCD TEM criteria by Shoemark et al).

#Non-hallmark TEM defects included class 2 defects such as central complex defects

*Low nNO according to accepted thresholds such as 77nL/min for tidal breathing

Table S2: Results of transmission electron microscopy of EPIC-PCD participants, overall and by age group (N=197)

	**Total N (%)**	**Age 0-6 y N (%)**	**Age 7-14 y N (%)**	**Age 15-30 y N (%)**	**Age 31-50 y N (%)**	**Age >50 y N (%)**
**Number of participants**	197 (100)	22 (100)	66 (100)	69 (100)	25 (100)	15 (100)
**Hallmark defects**[+]						
ODA & IDA-defect	62 (31)	10 (45)	17 (26)	27 (40)	6 (24)	2 (13)
ODA-defect	27 (13)	1 (5)	12 (18)	10 (14)	2 (8)	2 (13)
Microtubular disorganisation & IDA defect	29 (15)	2 (9)	10 (15)	10 (14)	2 (8)	5 (34)
**Non-hallmark defects**[#]						
Central complex defect	17 (9)	0 (0)	3 (4)	6 (9)	7 (28)	1 (7)
Other	21 (11)	0 (0)	9 (14)	7 (10)	3 (12)	2 (13)
Normal ultrastructure	41 (21)	9 (41)	15 (23)	9 (13)	5 (20)	3 (20)

EPIC-PCD: Ear-nose throat prospective international cohort of patients with primary ciliary dyskinesia. y: years. Results presented as N and column %. ODA: outer dynein arm. IDA: inner dynein arm defect.

[+]Hallmark TEM defects: ODA, ODA and IDA, microtubular disorganisation and IDA defects (based on BEAT-PCD TEM criteria by Shoemark et al).

[#]Non-hallmark TEM defects included class 2 defects such as central complex defects

Table S3: Upper respiratory symptoms of past 3 months reported by EPIC-PCD participants with positive PCD diagnosis (bi-allelic mutation or hallmark TEM defect) (N=257)

	**PCD positive N (%)**
**Number of participants**	257 (100)
**Nasal symptoms**	
Daily/often	133 (52)
Sometimes/rarely	97 (38)
Never	27 (11)
**Nasal symptoms persisting all the time**[a]	94 (55)
**Type of nasal symptoms**[b]	
Rhinorrhoea	203 (88)
Blocked nose	155 (67)
Sneezing	53 (23)
Anosmia/hyposmia	35 (15)
**Colour of nasal discharge in case of rhinorrhoea**[c]	
Clear	43 (21)
White	40 (20)
Yellow	70 (35)
Green	42 (21)
Mixed with blood	8 (4)
**Snoring**	
Daily/ often	37 (14)
Sometimes/rarely	90 (35)
Never/ not reported	130 (51)
**Periods of snoring**[d]	
Almost every night	35 (28)
Only during colds	54 (42)
Sometimes also without colds	29 (23)
Not reported	9 (7)
**Headache**	
Daily/often	27 (10)
Sometimes/rarely	130 (51)
Never/ not reported	100 (39)
**Headache when bending down**	25 (10)
**Migraines**	
Yes	21 (8)
No	236 (92)
**SNOT–22 completed**	99 (39)
Score median (IQR)	32 (23-42)

EPIC-PCD: Ear-nose throat prospective international cohort of patients with primary ciliary dyskinesia. Hallmark TEM (transmission electron microscopy) defects: outer dynein arm defect (ODA), ODA and inner dynein arm defect (IDA), microtubular disorganisation and IDA defect. Symptoms are presented as N and column %. SNOT-22 presented as median and IQR: interquartile range. Sino Nasal Outcome Test-22 (SNOT–22) questionnaire on chronic rhinosinusitis related items scored 0–5 ("No problem" to "Problem as bad as it can be"), total score range 0–110, mild 0–20, moderate 21–50, severe ≥51. [a]: Among 230 people with chronic nasal symptoms [b]: Among 230 people with chronic nasal symptoms, categories are not exclusive. [c]: Among 203 people with rhinorrhoea, categories are not exclusive. [d]: Among 127 people with snoring.

Table S4: Sinonasal examination results of EPIC-PCD participants with positive PCD diagnosis (bi-allelic mutation or hallmark TEM defect) (N=251)

	**PCD positive N (%)**
**ENT consultations on site**	251 (100)
**Nose appearance**	
Normal	133 (53)
Blocked	111 (44)
Not recorded	7 (3)
**Nasal discharge present**	
Yes	181 (72)
No	63 (25)
Not recorded	7 (3)
**Type of nasal discharge[a]**	
Serous	55 (30)
Sero-mucous	85 (47)
Muco-purulent	35 (19)
Mixed with blood	1 (1)
Not recorded	5 (3)
**Nasal mucosa**	
Abnormal	113 (45)
Normal	128 (51)
Not recorded	10 (4)
**Nasal polyps**	
Yes	36 (14)
No	193 (77)
Not assessed	22 (9)
**Nasal polyps size[bc]**	
Fully blocking	7 (19)
Partially blocking	27 (75)
Not assessed	2 (6)
**Bilaterally[bc]**	
Fully blocking	3 (8)
Partially blocking	12 (33)
Not recorded	21 (59)
**Unilaterally[bc]**	
Fully blocking	4 (11)
Partially blocking	19 (53)
Not recorded	13 (36)

EPIC-PCD: Ear-nose throat prospective international cohort of patients with primary ciliary dyskinesia. Hallmark TEM (transmission electron microscopy) defects: outer dynein arm defect (ODA), ODA and inner dynein arm defect (IDA), microtubular disorganisation and IDA defect. Examination findings are presented as N and column %. [a]: Among 181 people with nasal discharge. [b]: Among 36 people with nasal polyps. [c]: Nasal polyps described as partially blocking or with Lildholdt score 1 or 2, fully blocking or with Lildholdt score 3.

Table S4 (continued): Sinonasal examination results of EPIC-PCD participants with positive PCD diagnosis (bi-allelic mutation or hallmark TEM defect) (N=251)

	**PCD positive N (%)**
**ENT consultations on site**	251 (100)
**Inferior nasal turbinates**	
Normal	140 (56)
Hypertrophy	87 (35)
Atrophy	3 (1)
Not recorded	21 (8)
**Deviated nasal septum**	
Yes	79 (31)
Bulging forward	4 (2)
No	148 (59)
Not recorded	20 (8)
**Facial pain or sensitivity**	
Yes	39 (16)
No	194 (77)
Not recorded	18 (7)

EPIC-PCD: Ear-nose throat prospective international cohort of patients with primary ciliary dyskinesia. Hallmark TEM (transmission electron microscopy) defects: outer dynein arm defect (ODA), ODA and inner dynein arm defect (IDA), microtubular disorganisation and IDA defect. Examination findings are presented as N and column %.

Table S5: Upper airway management of EPIC-PCD participants, overall and by age group (N=384)

	Total N (%)	Age 0-6 y N (%)	Age 7-14 y N (%)	Age 15-30 y N (%)	Age 31-50 y N (%)	Age >50 y N (%)	*p*-value[a]
**Number of participants**	384 (100)	44 (100)	122 (100)	153 (100)	42 (100)	23 (100)	
**Hospitalisation since last consultation**	76 (20)	7 (16)	17 (14)	25 (16)	18 (43)	9 (39)	<0.001
Not recorded	308 (80)	37 (84)	105 (86)	128 (84)	24 (57)	14 (61)	
For sinonasal surgeries	9 (2)	0 (0)	3 (2)	2 (1)	3 (7)	1 (4)	0.202
For other surgeries	11 (3)	0 (0)	2 (2)	4 (3)	0 (0)	5 (22)	<0.001
i.v. antibiotics[b]	45 (12)	2 (5)	10 (8)	15 (10)	13 (31)	5 (22)	0.004
Further testing/ annual check-up	11 (3)	5 (11)	2 (2)	4 (3)	0 (0)	0 (0)	0.014
**Antibiotics prescribed for infection of**							0.019
upper respiratory tract	17 (4)	1 (2)	6 (5)	10 (7)	0 (0)	0 (0)	
lower respiratory tract	71 (19)	9 (20)	27 (22)	23 (15)	9 (21)	3 (13)	
other	20 (5)	2 (5)	3 (2)	5 (3)	6 (15)	4 (17)	
Not recorded	276 (72)	32 (73)	86 (71)	115 (75)	27 (64)	16 (70)	
**Nasal corticosteroids**							<0.001
Yes	82 (21)	3 (7)	19 (15)	34 (22)	17 (40)	9 (39)	
No	249 (64)	33 (75)	91 (75)	100 (66)	18 (43)	4 (17)	
Not recorded	56 (15)	8 (18)	12 (10)	19 (12)	7 (17)	10 (44)	
During all year	69 (18)	1 (2)	17 (13)	30 (20)	13 (31)	8 (35)	0.013
During exacerbations	11 (3)	2 (5)	2 (2)	4 (3)	3 (7)	0 (0)	0.374
**Nasal rinsing**							<0.001
Yes	187 (49)	11 (25)	63 (50)	80 (51)	23 (54)	10 (34)	
No	110 (28)	20 (45)	44 (39)	40 (27)	4 (9)	2 (9)	
Not recorded	87 (23)	13 (30)	15 (11)	33 (22)	15 (37)	11 (47)	
During all year	154 (40)	10 (23)	55 (45)	64 (42)	17 (40)	8 (35)	0.536
During exacerbations	20 (5)	1 (2)	5 (4)	9 (6)	3 (7)	2 (9)	0.692
**Regular nose blowing**							<0.001
Yes	164 (43)	17 (39)	63 (52)	53 (35)	21 (50)	10 (44)	
No	118 (31)	12 (27)	40 (33)	60 (39)	5 (12)	1 (4)	
No recorded	102 (26)	15 (34)	19 (15)	40 (26)	16 (48)	12 (52)	
During all year	149 (39)	16 (36)	61 (50)	47 (31)	17 (40)	8 (35)	0.315
During exacerbations	5 (1)	0 (0)	1 (1)	3 (2)	1 (2)	0 (0)	0.744
**Nebulisation**							<0.001
Yes	46 (12)	2 (5)	16 (13)	15 (10)	10 (23)	3 (13)	
No	212 (55)	26 (59)	80 (66)	88 (58)	12 (29)	6 (26)	
Not recorded	126 (33)	16 (36)	26 (21)	50 (32)	20 (48)	14 (61)	
During all year	43 (11)	2 (5)	15 (12)	15 (10)	8 (19)	3 (13)	0.279
During exacerbations	0 (0)	0 (0)	0 (0)	0 (0)	0 (0)	0 (0)	
Isotonic saline	17 (4)	0 (0)	6 (5)	4 (3)	6 (14)	1 (4)	0.025
Hypertonic saline	22 (6)	2 (5)	7 (6)	9 (6)	3 (7)	1 (4)	0.989

EPIC-PCD: Ear-nose throat prospective international cohort of patients with primary ciliary dyskinesia. y: years. Isotonic saline: NaCl 0.9%. Hypertonic saline: NaCl >0.9%. [a]: Chi-square test of independence. [b]: i.v. antibiotics for acute pulmonary exacerbations or Pseudomonas eradication.

Table S6: Association of EPIC-PCD study centre with sinonasal disease (N=384)

	Number of participants (%)	Odds ratio	95% confidence interval
**Study centres**	384 (100)		
Paris	52 (14)		**Reference**
Amsterdam	26 (7)	0.19	0.04–0.88
Ankara	60 (16)	0.57	0.25–1.32
Berlin	43 (11)	1.28	0.56–2.93
Bern	7 (2)	0.00	0.00–0.00
Cyprus	21 (5)	3.23	1.21–8.63
Istanbul	58 (15)	2.76	1.29–5.92
Leuven	11 (3)	2.74	1.29–5.92
Liège	10 (3)	3.59	0.99–13.06
Oslo	38 (10)	0.60	0.23–1.56
Southampton	42 (10)	0.30	0.10–0.90
Valencia	16 (4)	0.55	0.14–2.16

Results of univariable ordinal logistic regression model, including only study centre as an explanatory variable (reference centre: Paris). Sinonasal disease defined with composite outcome score consisting of three variables: patient-reported headache while bending down as a proxy for sinusitis, and ENT examination findings of nasal polyps and facial pain. EPIC-PCD: Ear-nose throat prospective international cohort of patients with primary ciliary dyskinesia.

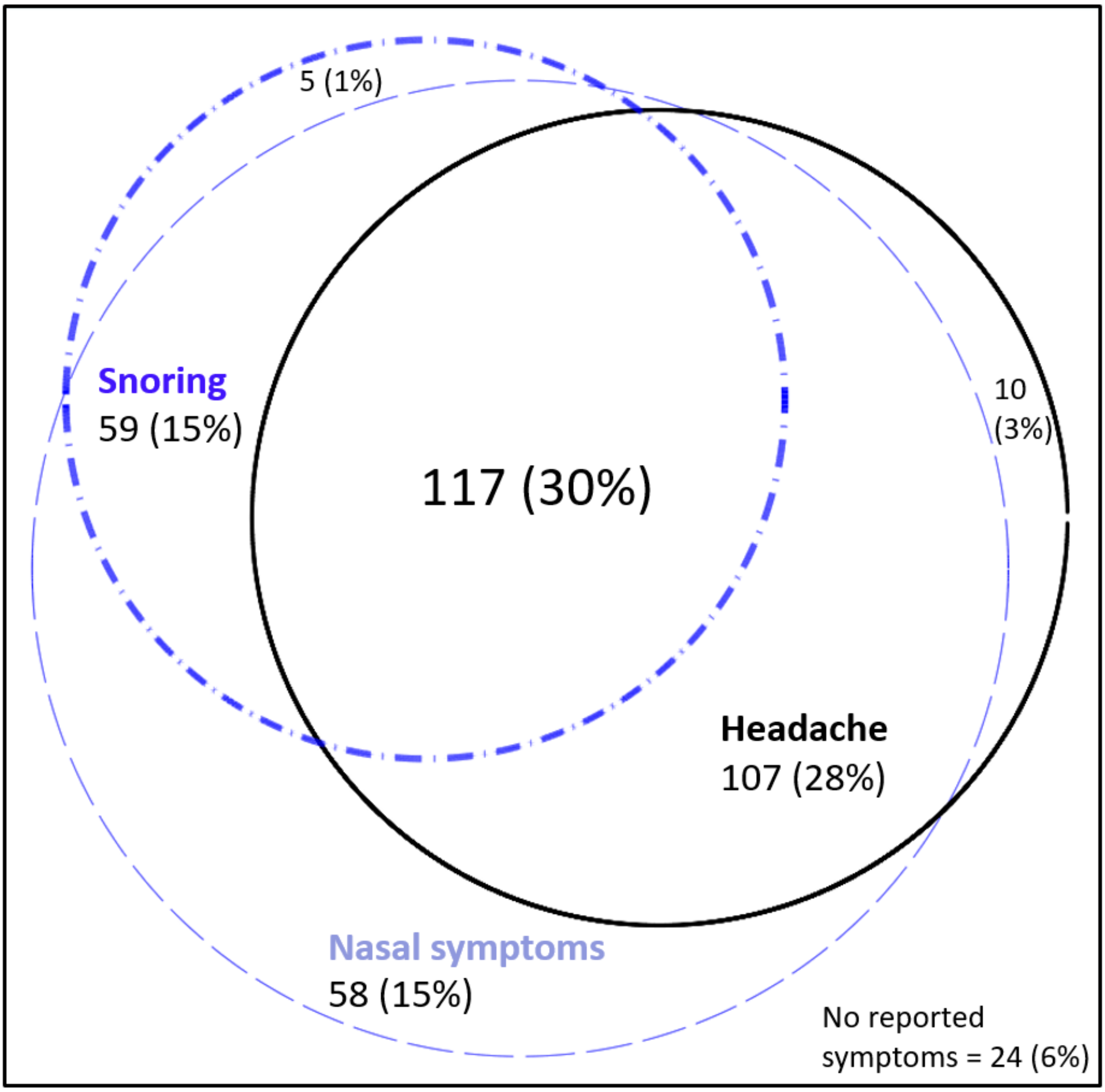

Figure S1: Proportion of patient-reported symptoms (snoring, headache, nasal symptoms) during the past three months among EPIC-PCD participants (N=384).

EPIC-PCD: Ear-nose throat prospective international cohort of patients with primary ciliary dyskinesia.

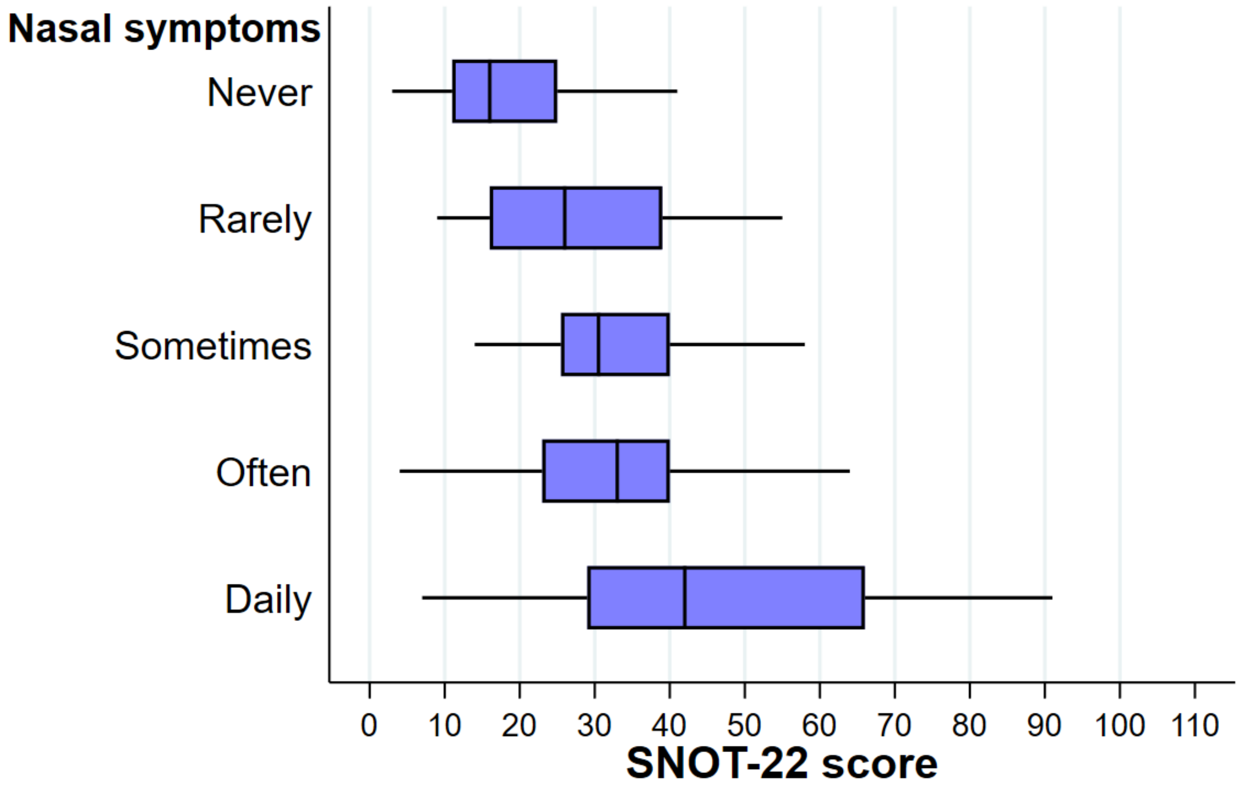

Figure S2: Sino-Nasal Outcome Test (SNOT)-22 score by frequency of patient-reported nasal symptoms during the past three months among EPIC-PCD participants (N=136).

SNOT-22 scores 0–5 (“No problem” to “Problem as bad as it can be”); total score range 0–110; mild 0–20, moderate 21–50; severe ≥ 51. Score ranges indicated by horizontal lines. EPIC-PCD: Ear-nose throat prospective international cohort of patients with primary ciliary dyskinesia.

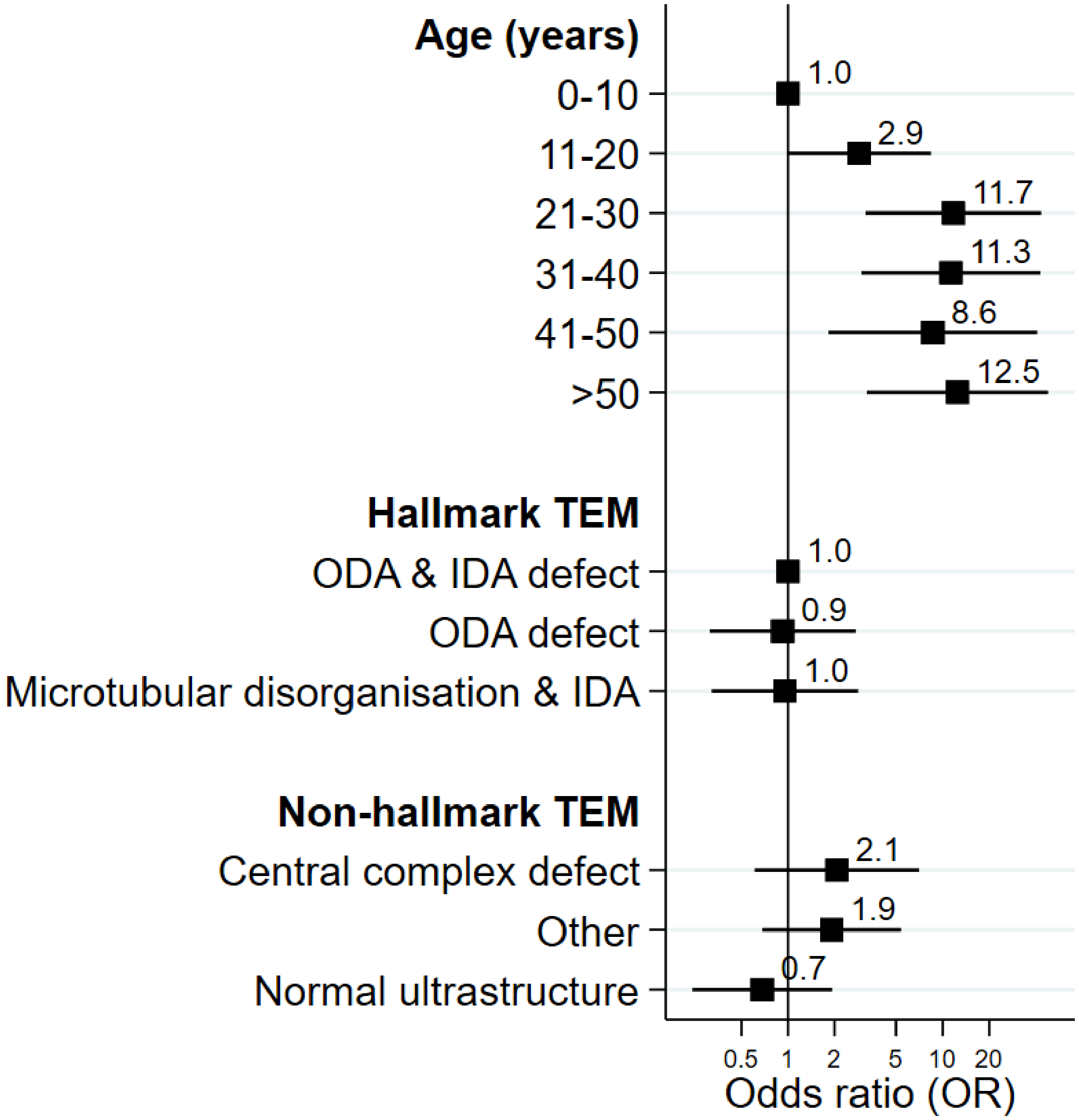

Figure S3: Association of age and ciliary ultrastructural defect with sinonasal disease in EPIC-PCD participants (N=197).

Sinonasal disease defined by composite outcome score consisting of three variables: patient-reported headache while bending down as a proxy for sinusitis, ENT examination findings of nasal polyps, and facial pain. EPIC-PCD: Ear-nose throat prospective international cohort of patients with primary ciliary dyskinesia. ODA: outer dynein arm. IDA: inner dynein arm. TEM: transmission electron microscopy. Odds ratio (OR) indicated by squares and 95% CI indicated by horizontal lines.
